# The influencing factors of health–related quality of life of the general population of Iran during the COVID-19 Pandemic

**DOI:** 10.3389/fmed.2023.1049642

**Published:** 2023-02-16

**Authors:** Maryam Shirvani Shiri, Hassan Karami, Hosein Ameri, Ali Akbari Sari, Maryam Tatari, Sara Emamgholipour, Somayeh Afshari

**Affiliations:** ^1^Department of Health Management and Economics, School of Public Health, Tehran University of Medical Sciences, Tehran, Iran; ^2^Department of Health Management and Economics, School of Health Management and Information Sciences, Iran University of Medical Sciences, Tehran, Iran; ^3^Department of Health Management and Economics, Health Policy and Management Research Center, School of Public Health, Shahid Sadoughi University of Medical Sciences, Yazd, Iran; ^4^Department of Epidemiology and Biostatistics, School of Public Health, Tehran University of Medical Sciences, Tehran, Iran; ^5^Vice Chancellery of Health, Torbat Heydariyeh University of Medical Sciences, Torbat-e Heydariyeh, Iran

**Keywords:** health–related quality of life, COVID-19, socioeconomic factors, regression analysis, pandemic, Iran

## Abstract

COVID-19 is a global challenge that negatively affects the health–related quality of life (HRQoL) of the general population. The current study aimed to evaluate HRQoL and its associated factors among the Iranian general population during the COVID-19 pandemic. The data were collected in 2021 using the EuroQol 5-Dimension 3-Level (EQ-5D-3L) and EQ-5D Visual Analog Scale (EQ VAS) questionnaires through an online survey. Participants were recruited *via* social media from the Fars province. The multiple binary logistic regression model was used to identify factors influencing participants' HRQoL. Kolmogorov-Smirnov, the *t*-test, ANOVA, and the chi-square test were used. All tests were conducted at a significance level of 5% using Stata 14.2 and SPSS 16. A total of 1,198 participants were involved in this cross-sectional study. The mean age of participants was 33.3 (SD:10.2), and more than half were women (55.6%). The mean EQ-5D-3L index value and EQ-VAS of the respondents were 0.80 and 77.53, respectively. The maximum scores of the EQ-5D-3L and EQ-VAS in the present study were 1 and 100, respectively. The most frequently reported problems were anxiety/depression (A/D) (53.7%), followed by pain/discomfort (P/D) (44.2%). Logistic regression models showed that the odds of reporting problems on the A/D dimension increased significantly with supplementary insurance, including concern about getting COVID-19, hypertension, and asthma, by 35% (OR = 1.35; *P* = 0.03), 2% (OR = 1.02; *P* = 0.02), 83% (OR = 1.83; *P* = 0.02), and 6.52 times (OR = 6.52; *P* = 0.01), respectively. The odds of having problems on the A/D dimension were significantly lower among male respondents, those in the housewives + students category, and employed individuals by 54% (OR = 0.46; *P* = 0.04), 38% (OR = 0.62; *P* = 0.02) and 41% (OR = 0.59; *P* = 0.03), respectively. Moreover, the odds of reporting a problem on the P/D dimension decreased significantly in those belonging in a lower age group and with people who were not worried about getting COVID-19 by 71% (OR = 0.29; *P* = 0.03) and 65% (OR = 0.35; *P* = 0.01), respectively. The findings of this study could be helpful for policy-making and economic evaluations. A significant percentage of participants (53.7%) experienced psychological problems during the pandemic. Therefore, effective interventions to improve the quality of life of these vulnerable groups in society are essential.

## Introduction

The new coronavirus (COVID-19) has spread rapidly worldwide. By the beginning of 2022, more than 300 million people had been infected globally, and about five million had died (1). In Iran, the first case of infection was reported on 19/02/2020. In January 2022, the number of confirmed cases of COVID-19 was over 6,373,174, and the number of deaths from COVID-19 was more than 132,454 (2). Around the world, a variety of strong social distancing measures have been implemented to slow the growth rate of COVID-19 cases (e.g., in Wuhan and other Chinese cities (3, 4), across European countries (5), French regions (6), or some U.S. states (7, 8).

Similarly, the Iranian government adopted strong measures such as closing down schools, universities, and workplaces and propagating strict social distancing to reduce the prevalence of COVID-19. Such restrictions increased long-term psychological consequences and negatively affected the quality of life (QoL) of individuals through fear and anxiety, stress, and stigmatization (9, 10).

The World Health Organization defines the quality of life as people's perception of their position in life in terms of culture, the value system in which they live, and their goals, expectations, standards, and priorities. Therefore, it is a completely subjective topic that cannot be observed by others and is based on people's understanding of different aspects of life. This term is a wide-ranging notion that encompasses, in a complex way, a person's physical health, psychological condition, level of independence, and relation to notable features of their environment (11, 12).

Health–related quality of life (HRQoL) refers to those aspects of QoL that influence either physical or mental health. This measure enables healthcare policymakers to identify the factors affecting HRQoL and recognize those aspects of COVID-19 management that need to be enhanced to improve people's HRQoL (13, 14).

One of the most widely used instruments for measuring HRQoL in clinical and outcome research is the EuroQol 5-Dimension 3-Level (EQ-5D-3L) (15), which contains a descriptive system of five dimensions (Mobility, Self-Care, Usual-Activity, Pain-Discomfort, and Anxiety-Depression) and an EQ-5D Visual Analog Scale (EQ VAS) (16). EQ-5D is a generic measure recommended by the National Institute of Health and Care Excellence (NICE) to calculate the utility values of health states (17). The Iranian value set of EQ-5D-3L was estimated based on the time trade-off (TTO) method by Goudarzi et al. (18).

According to the available literature, the HRQoL of the general population is influenced by several socioeconomic and clinical factors. In a population-wide study, Ping et al. concluded that factors such as aging, chronic disease, lower income, epidemic effects, and concern about getting COVID-19 are effective in affecting HRQoL (19). Moreover, an Estonian study reported that being older, unemployed or economically inactive, and experiencing financial hardship were all correlated with lower HRQoL (20). Regarding the wide range of reports on HRQoL in the general population during the COVID-19 pandemic in other countries, such as China (19), Portugal (21), Vietnam (22), Egypt (23), Estonia (20), and Saudi Arabia (24), the lack of extensive national and subnational scale studies on the subject, the scarcity of studies on the relationship between HRQoL and socioeconomic and clinical factors, the assessment of the HRQoL of the Iranian general population and the identification of influential predictors of HRQoL during the COVID-19 pandemic need to be investigated. As a result, the present study evaluated the HRQoL of the general population during the COVID-19 pandemic and its relationship with socioeconomic and clinical factors in the Fars province, southern Iran.

## Methods

### Study design and context

This cross-sectional research was conducted on the general population in the Fars province from 23/10/2021 to 21/11/2021 (during the fifth wave of COVID-19). Fars is the fourth most populated province (4,851,274 people) in Iran. It is located in the south of the country and includes 36 cities (25). By 31/01/2022, a total of 534,127 confirmed COVID-19 cases with 7,485 deaths had been reported in the province (2).

### Sample size

The sample size of this study was calculated by the$n=\frac{z_{1-\frac{a}{2}}^{2}\sigma^{2}}{d^{2}}$ formula at a 95% confidence level (α−1), σ = 19.37, and the acceptable margin of error for the d parameter was 1.2 (19). The sample size was increased by 10% based on the probability of losing the number of samples during the study, and the final sample size was estimated to be 1,146 participants. Participants were recruited through convenience sampling.

### Study participants

The study population was comprised of inhabitants of the Fars province. Inclusion criteria were: (1) being 18 years of age or older; (2) having access to the internet and the online questionnaire; (3) agreeing to participate in the study by confirming the online consent form; and (4) having the complete ability to answer all questionnaires. Furthermore, people who were not residents of the Fars province, COVID-19 patients, in addition to those who were previously affected by COVID-19, and individuals with a past medical history of mental illness or who were under treatment for a mental health problem were excluded.

### Definition of variables

HRQoL was the response variable, and the explanatory variables included socio-economic and clinical factors such as gender (female respondents vs. male respondents); marital status (single vs. married); age (≤ 30, 31–40, 41–50, and ≥51 years); educational level (illiterate; <6th grade; 6–9th grade; 10–12th grade; >12th grade); employment status (employed; housewives+students; unemployed); insurance coverage (no vs. yes); place of residence (urban vs. rural); level of household income (very low; low; middle; high); concern about getting COVID-19 (never; no; yes; very worried), hypertension (no vs. yes); diabetes (no vs. yes); cardiovascular disease (no vs. yes); cerebrovascular disease (no vs. yes); pulmonary disease (no vs. yes); asthma (no vs. yes).

### Procedure

The current study was a web-based survey, and respondents participated in it *via* instant messaging (WhatsApp and Telegram). We found all the administrators of Telegram and WhatsApp channels by searching on Google and introducing ourselves through friends. We contacted the administrators of the Telegram and WhatsApp channels for all the cities in the Fars province and asked them to post the link to the online questionnaires for this study in their groups and invite members to complete the questionnaires. This included a statement outlining the objectives of the research and informed consent to participate in the study. Following confirmation of these statements, participants proceeded to the main stage of the questionnaire.

The data collection tool was a questionnaire consisting of the following sections:

1. Demographic and socio-economic information of participants.
2. Health status: health status variables include chronic diseases and behaviors associated with the COVID-19 pandemic. This information was self-reported. The behavior related to the pandemic was also defined as the degree of concern about being infected by COVID-19; responses were categorized into four states: “I'm never worried,” “I'm not worried,” “I'm slightly worried,” and “I'm very worried.”
3. The 3-Level version of the EuroQol 5-Dimension questionnaire (EQ-5D-3L) and the Visual Analog Scale (VAS) (validated Farsi version of the HRQoL questionnaire): this was used to determine the health status of participants. The EQ-5D-3L questionnaire consists of five questions, each measuring one of the five dimensions of HRQoL: Mobility (MO), Self-Care (SC), Usual Activities (UA), Pain/Discomfort (P/D), and Anxiety/Depression (A/D). The questions in each dimension are answered on a three-level scale, including no problems, some problems, and extreme problems. The scales were given a score from 1 (no problems) to 3 (extreme problems). Eventually, a five-digit code was obtained for each patient by putting the scores' numbers together. This method can generate 243 unique discrete health states (five to the power of three). The EuroQOL Group performed research mainly focusing on statistical modeling to produce numerical values for each of the 243 health states obtained from the EQ-5D-3L questionnaire. This utility-based EQ-5D-3L index score ranges from−0.113 (most severe impairment across all five dimensions) to 1 (no problems on any dimension) (18). EQ VAS is another part of the instrument that measures an individual's personal view of their HRQoL using a scale of 0 (worst health state) to 100 (best health state). This tool can be used to quantitatively assess respondents' health outcomes (26). The validity and reliability of the EQ-5D-3L were confirmed by weighted kappa coefficients of 0.66 to 0.92 and ICCs of 0.88 for cancer patients (27), in addition to kappa coefficients of 0.39 to 0.71, ICCs of 0.76, and the Cronbach's alpha of 0.87 for patients with type 2 diabetes in Iran (28).

### Statistical analysis

Continuous variables were represented as mean ± standard deviation and categorical variables as frequencies and percentages. Since the result of the Kolmogorov–Smirnov normal distribution test for the EQ-5D-3L was significant (*p* < 0.05), the *T-*test and ANOVA tests were used to determine the differences in the EQ-5D-3L index value in each factor. For each dimension of the EQ-5D-3L, the second and third levels were merged to create two broader levels: “no problems” and “some or extreme problems.” Then, the chi-square test was used to assess the relationship between the EQ-5D-3L dimensions and qualitative variables. Finally, multiple logistic regression was used to obtain odds ratios (ORs) and 95% confidence intervals (95% CIs) for variables that were significantly associated with dimensions in the chi-square test. Key assumptions of multiple logistic regression were met. The independence of errors was not violated (Durbin-Watson statistic = 2.03). Also, multicollinearity was met (variance inflation factOR = ≤ 1.5). Furthermore, variables were entered into the model using the backward elimination technique. All tests were conducted using Stata 14.2 (StataCorp, College Station, TX) and SPSS 16 software at a significance level of 5%.

## Results

### Demographic characteristics

A total of 1,198 questionnaires were completed and returned by the participants by 21/11/2021. A few cases (amounting to 32) were not usable due to living outside of Fars's province. The remaining 1,166 questionnaires were analyzed.

The sociodemographic and clinical characteristics of 1,166 participants are shown in Table 1. The mean age of participants was 33.3 (SD:10.2). More than half of the participants were women (55.6%), married (67.6%), urbanites (82.8%), and highly educated (65.2%). The mean± SD for the EQ-5D-3L Index and EQ-VAS were 0.80 ± 0.016 and 77.53 ± 21.29, respectively (Table 1).

**Table 1:** Demographic characteristics.

**Variables**	**Category**	**Number**	**Percentage**
Gender	Female	648	55.6
	Male	518	44.4
Age group	≤ 30	423	36.3
	31–40	483	41.4
	41–50	195	16.7
	≥51	53	4.5
Marital status	Single	378	32.4
	Married	788	67.6
Urbanization	Urban	966	82.8
	Rural	200	17.2
Employment status	Employed	609	52.2
	Housewives + students	460	39.5
	Unemployed	97	8.3
Education	Illiterate	7	0.6
	> 6th grade	25	2.1
	6–9th grade	85	7.3
	10–12th grade	289	24.8
	>12th grade	760	65.2
Income level	Very low	87	7.5
	Low	317	27.2
	Middle	716	61.4
	High	46	3.9
Insurance	No	221	19.0
	Yes	945	81.0
Supplementary insurance	No	763	65.4
	Yes	403	34.6
Concern about contracting COVID-19	Never	68	5.8
	No	205	17.6
	Yes	597	51.2
	Very concerned	292	25.0
Hypertension	No	1,112	95.4
	Yes	54	4.6
Diabetes	No	1,138	97.6
	Yes	28	2.4
Cardiovascular disease	No	1,146	98.3
	Yes	20	1.7
Cerebrovascular disease	No	1,161	99.6
	Yes	5	0.4
Pulmonary disease	No	1,155	99.1
	Yes	11	0.9
Asthma	No	1,138	97.6
	Yes	28	2.4
EQ-5D 3L index	Mean: 0.80		SD: 0.17
EQ-VAS	Mean: 77.53		SD: 21.30

SD, Standard deviation; EQ-5D 3L, EuroQol 5-Dimension 3-Level questionnaire; EQ-VAS, EuroQol-Visual Analog Scale.

### EQ-5D-3L index values for each variable

The results of the differences in EQ-5D-3L index values for each factor are presented in Table 2. The parametric tests demonstrated that the differences in EQ-5D-3L index values were statistically significant (*P* < 0.001) for education, occupation, income, worry about COVID-19, hypertension, asthma, cerebrovascular disease, and pulmonary disease. The results also showed that the mean EQ-5D-3L value was significantly lower in participants with a lower level of income (0.69 ± 0.20) vs. those with a higher level of income (0.82 ± 0.21), in the illiterate (0.65 ± 0.25) vs. those with higher levels of education (0.81 ± 0.15), in unemployed participants (0.75 ± 0.18) vs. those in employment (0.82 ± 0.15). The mean EQ-5D-3L value was also lower in participants who were worried about COVID-19 (0.74 ± 0.18) vs. those who never worried about COVID-19 (0.82 ± 0.17), those with hypertension (0.72 ± 0.19) vs. those without (0.80 ± 0.16), in respondents diagnosed with a cerebrovascular disease (0.66 ± 0.08) vs. those without (0.80 ± 0.16), in those with pulmonary disease (0.62 ± 0.13) vs. those without (0.80 ± 0.16), and in individuals with asthma (0.68 ± 0.13) vs. those without such a diagnosis (0.80 ± 0.16) (Table 2).

**Table 2:** Differences in EQ-5D-3L index values among participants.

**Variables**	**Category**	**Mean±SD**	***P***
Gender	Female	0.78 ± 0.16	0.241^*^
	Male	0.81 ± 0.16		
Age group	≤ 30	0.81 ± 0.16	0.171^†^
	31–40	0.80 ± 0.16	
	41–50	0.78 ± 0.16	
	≥51	0.73 ± 0.18	
Marital status	Single	0.81 ± 0.16	0.142^*^
	Married	0.79 ± 0.16	
Urbanization	Urban	0.79 ± 0.16	0.370^*^
	Village	0.80 ± 0.17	
Levels of education	Illiterate	0.65 ± 0.25	**0.011** ^†^
	<6th grade	0.74 ± 0.20	
	6–9th grade	0.76 ± 0.19	
	10–12th grade	0.78 ± 0.17	
	12th grade <	0.81 ± 0.15	
Employment status	Employed	0.82 ± 0.15	**0.006** ^†^
	Housewives + students	0.79 ± 0.17	
	Unemployed	0.75 ± 0.18	
Income levels	Very low	0.69 ± 0.20	**<0.001** ^†^
	Low	0.77 ± 0.16	
	Middle	0.82 ± 0.15	
	High	0.82 ± 0.21	
Insurance	Yes	0.80 ± 0.16	0.761^*^
	No	0.78 ± 0.18	
Supplementary insurance	Yes	0.81 ± 0.16	0.122^*^
	No	0.79 ± 0.17	
Concern about contracting COVID-19	Never	0.82 ± 0.17	**<0.001** ^†^
	No	0.86 ± 0.17	
	Yes	0.80 ± 0.15	
	Very concerned	0.74 ± 0.18	
Hypertension	Yes	0.72 ± 0.19	**0.012** ^*^
	No	0.80 ± 0.16	
Diabetes	Yes	0.75 ± 0.18	0.861^*^
	No	0.80 ± 0.16	
Cardiovascular disease	Yes	0.76 ± 0.17	0.133^*^
	No	0.80 ± 0.16	
Cerebrovascular disease	Yes	0.66 ± 0.08	**0.005** ^*^
	No	0.80 ± 0.16	
Pulmonary disease	Yes	0.62 ± 0.13	**0.041** ^*^
	No	0.80 ± 0.16	
Asthma	Yes	0.68 ± 0.13	**<0.001** ^*^
	No	0.80 ± 0.16	

EQ-5D 3L, EuroQol 5-Dimension—Level questionnaire; SD, Standard Deviation; Boldness: P < 0.05.

* Statistical significance of differences calculated using t-test.

† Statistical significance of differences calculated using ANOVA.

### EQ-5D dimensions

Table 3 shows the result of the chi-square test between dimensions dichotomized (dependent variables) and qualitative variables (independent variables). Of the total respondents, 21.5, 7.2, 16, 44.2, and 53.7% reported problems in the dimensions of MO, SC, UA, P/D, and A/D, respectively. People over the age of 51 and married reported significantly more problems on the MO dimension (*P* < 0.05). Illiterate people and those without supplementary insurance reported the most problems on the SC dimension (*P* < 0.05). Male respondents, people over the age of 51, unemployed individuals, and those with hypertension reported the most problems in the UA dimension (*P* < 0.05). Female respondents, people with < 6 years of schooling, unemployed individuals, people without health insurance, and those with hypertension and asthma reported the most problems on the P/D dimension (*P* < 0.05). Female interviewees, unemployed people, those without health insurance, individuals with supplementary insurance, and subjects with hypertension and asthma reported the most problems on the A/D dimension (*P* < 0.05). People with very low incomes reported the most problems across all dimensions (*P* < 0.05). Subjects who were very concerned about getting COVID-19 reported the most problems across all dimensions except the A/D dimension (*P* < 0.05). Moreover, people with a pulmonary disease diagnosis reported the most problems across all dimensions except the MO dimension (*P* < 0.05) (Table 3).

**Table 3:** Results of the chi-square test for the EQ-5D-3L dimensions and qualitative variables.

**Variables**	**Category**	**Frequency (Percentages) with any problems:** ***N*** **(%)**				
		**Mobility**	**Self-care**	**Usual activities**	**Pain/** **Discomfort**	**Anxiety/** **Depression**
Overall, with problems		251 (21.5)	84 (7.2)	187 (16.0)	515 (44.2)	626 (53.7)
Gender	Female	138 (21.3)	40 (6.2)	87 (13.4)	**319 (49.2)**	**400 (61.7)**
	Male	113 (21.8)	44 (8.5)	**100 (19.3)**	196 (37.8)	226 (43.6)
Age group	≤ 30	75 (17.7)	25 (5.9)	62 (14.7)	169 (40.0)	220 (52.0)
	31–40	103 (21.3)	34 (7.0)	67 (13.9)	205 (42.4)	258 (53.4)
	41–50	50 (25.6)	18 (9.2)	35 (17.9)	98 (50.3)	111 (56.9)
	≥51	**23 (35.4)**	7 (10.8)	**23 (35.4)**	43 (66.2)	37 (56.9)
Marital status	Single	187 (23.7)	60 (7.6)	136 (17.3)	372 (47.2)	436 (55.3)
	Married	**64 (16.9)**	24 (6.3)	51 (13.5)	143 (37.8)	190 (50.3)
Urbanization	Urban	210 (21.7)	70 (7.2)	150 (15.5)	419 (43.4)	111 (55.5)
	Village	41 (20.5)	14 (7.0)	37 (18.5)	96 (48.0)	515 (53.3)
Levels of education	Illiterate	3 (42.9)	**1 (14.3)**	2 (28.6)	5 (71.4)	5 (71.4)
	<6th grade	8 (32.0)	3 (12.0)	7 (28.0)	**17 (68.0)**	12 (48.0)
	6–9th grade	21 (24.7)	11 (12.9)	20 (23.5)	41 (48.2)	50 (58.8)
	10–12th grade	66 (22.8)	27 (9.3)	43 (14.9)	149 (51.6)	158 (54.7)
	>12 grade	153 (20.1)	42 (5.5)	115 (15.1)	303 (39.9)	401 (52.8)
Employment status	Employed	127 (20.9)	44 (7.2)	94 (15.4)	243 (39.9)	293 (48.1)
	Housewives + students	98 (21.3)	36 (7.8)	67 (14.6)	215 (46.7)	270 (58.7)
	Unemployed	26 (26.8)	4 (4.1)	**26 (26.8)**	**57 (58.8)**	**63 (64.9)**
Income levels	Very low	**29 (33.3)**	**16 (18.4)**	**23 (26.4)**	**55 (63.2)**	**61 (70.1)**
	Low	74 (23.3)	25 (7.9)	59 (18.6)	165 (52.1)	194 (61.2)
	Middle	141 (19.7)	38 (5.3)	98 (13.7)	279 (39.0)	350 (48.9)
	High	7 (15.2)	5 (10.9)	7 (15.2)	16 (34.8)	21 (45.7)
Insurance	Yes	212 (22.4)	60 (6.3)	153 (16.2)	403 (42.6)	494 (52.3)
	No	39 (17.6)	**24 (10.9)**	34 (15.4)	**112 (50.7)**	**132 (59.7)**
Supplementary insurance	Yes	79 (19.6)	24 (6.0)	122 (16.0)	341 (44.7)	**433 (56.7)**
	No	172 (22.5)	60 (7.9)	65 (16.1)	174 (43.2)	193 (47.9)
Concern about contracting COVID-19	Never	13 (19.1)	5 (7.4)	9 (13.2)	24 (35.3)	31 (45.6)
	No	27 (13.2)	11 (5.4)	19 (9.3)	61 (29.8)	74 (36.1)
	yes	127 (21.3)	36 (6.0)	93 (15.6)	266 (44.6)	343 (57.5)
	Very concerned	**84 (28.8)**	**32 (11.0)**	**66 (22.6)**	**163 (55.8)**	177 (60.6)
Hypertension	Yes	15 (27.8)	7 (13.0)	**16 (29.6)**	**34 (63.0)**	**37 (68.5)**
	No	236 (21.2)	77 (6.9)	171 (15.4)	481 (43.3)	589 (53.0)
Diabetes	Yes	8 (28.6)	4 (14.3)	7 (25.0)	16 (57.1)	17 (60.7)
	No	243 (21.4)	80 (7.0)	180 (15.8)	499 (43.8)	609 (53.5)
Cardiovascular disease	Yes	4 (20.0)	1 (5.0)	6 (30.0)	13 (65.0)	12 (60.0)
	No	247 (21.6)	83 (7.2)	181 (15.8)	502 (43.8)	614 (53.6)
Cerebrovascular disease	Yes	2 (40.0)	0 (0)	1 (20.0)	5 (100.0)	5 (100.0)
	No	249 (21.4)	84 (7.2)	186 (16.0)	510 (43.9)	621 (53.5)
Pulmonary disease	Yes	4 (36.4)	**3 (27.3)**	**6 (54.5)**	**9 (81.8)**	**10 (90.9)**
	No	247 (21.4)	81 (7.0)	181 (15.7)	506 (43.8)	616 (53.3)
Asthma	Yes	9 (32.1)	3 (10.7)	5 (17.9)	**22 (78.6)**	**25 (89.3)**
	No	242 (21.3)	81 (7.1)	182 (16.0)	493 (43.3)	601 (52.8)

EQ-5D-3L, EuroQol 5-Dimensional 3Level; Bold values are statistically significant, P < 0.05.

### Factors associated with EQ-5D dimensions

Multiple logistic regression models were conducted to evaluate the relationships between the significant variables obtained from Table 3 (i.e., gender, age, employment status, income level, insurance coverage, concern about getting COVID-19, hypertension, and asthma) and EQ-5D-3L dimensions. As presented in Table 4, male respondents had higher odds of 73% (OR = 1.73; *P* = 0.03) to report a problem on the UA dimension compared to the female subjects (reference group), while they reported significantly fewer problems on the A/D dimension by 54% (OR = 0.46; *P* = 0.04). Compared to the age group of 50 years and over (reference group), the age group ≤ 30 reported a lower percentage of problems on the dimensions of MO= 64% (OR = 0.36; *P* = 0.01), SC= 69% (OR = 0.31; *P* = 0.03), UA= 66% (OR = 0.34; *P* = 0.01), and P/D= 69% (OR = 0.31; *P* = 0.01). The odds of reporting problems on the SC dimension increased by 2.56 (OR = 2.56; *P* = 0.02) and 3.1 (OR = 3.1; *P* = 0.01) times, respectively, for those in employment and housewives + students, while being employed and housewives + students significantly decreased the odds of reporting problems on the A/D dimension by 41% (OR = 0.59; *P* = 0.03) and 38% (OR = 0.62; *P* = 0.02), respectively, compared to the unemployed (reference group). In comparison with high-income people (reference group), those with very low income, low income, and middle income significantly increased the odds of a problem on the MO dimension by 2.78 times (OR = 2.78; *P* = 0.01), 72% (OR = 1.72; *P* = 0.02), and 39% (OR = 1.39; *P* = 0.02), respectively. Moreover, the odds of problems on the SC dimension among people with supplementary insurance were lower by 83% (OR = 0.17; *P* = 0.04), while the odds of reporting risk on the A/D dimension were higher by 35% (OR = 1.35; *P* = 0.03). Additionally, people who were not worried about getting COVID-19 had significantly lower odds in MO=60% (OR = 0.40; *P* = 0.01), UA= 68% (OR = 0.32; *P* = 0.01), P/D= 65% (OR = 0.35; *P* = 0.01), and A/D= 58% (OR = 0.42; *P* = 0.02). Moreover, the odds of reporting problems on the A/D dimension increased significantly by 83% and 6.52 times, respectively, in subjects with hypertension (OR = 1.83; *P* = 0.02) and asthma (OR = 6.52; *P* = 0.01).

**Table 4:** Results of multiple logistic regression for the EQ-5D-3L dimensions and qualitative variables.

**Variables**	**Category**	**Mobility**		**Self-care**		**Usual activities**		**Pain/Discomfort**		**Anxiety/Depression**	
		**A** ^*^ **OR**	**CI (95%)**	**AOR**	**CI (95%)**	**AOR**	**CI (95%)**	**AOR**	**CI (95%)**	**AOR**	**CI (95%)**
Gender	Male	1.15	(0.82,1.61)	1.82	(1.02,3.24)	**1.73**	**(1.18,2.5)**	0.59	(0.44,0.78)	**0.46**	**(0.35,0.62)**
	Female	Ref									
Age Groups	≤ 30	**0.36**	**(0.21,0.65)**	**0.31**	**(0.12,0.81)**	**0.34**	**(0.18,0.61)**	**0.31**	**(0.17,0.57)**	0.61	(0.32,1.17)
	31-40	**0.45**	**(0.26,0.80)**	**0.44**	**(0.18,1.08)**	**0.31**	**(0.17,0.56)**	**0.29**	**(0.16,0.52)**	0.7	(0.38,1.29)
	41-50	**0.60**	**(0.33,1.1)**	**0.66**	**(0.25,1.72)**	**0.39**	**(0.20,0.75)**	**0.38**	**(0.20,0.73)**	1	(0.53,1.88)
	≥ 51	Ref									
Employment status	Employed	0.88	(0.51,1.50)	**2.56**	**(0.85,7.65)**	0.7	(0.4,1,22)	0.73	(0.45,1,2)	**0.59**	**(0.37,0.95)**
	Housewives+ students	0.97	(0.54,1.71)	**3.10**	**(1.02,9.44)**	0.81	(0.44,1.48)	0.71	(0.43,1.19)	**0.62**	**(0.38,1.01)**
	Unemployed	Ref									
Income level	Very low	**2.78**	**(1.09,7.08)**	1.75	(0.55,5.56)	2.15	(0.79,5.86)	2.53	(1.11,5.73)	1.97	(0.88,4.39)
	Low	**1.72**	**(0.73,4.08)**	0.57	(0.19,1.65)	1.27	(0.51,3.11)	1.9	(0.94,3.86)	1.6	(0.82,3.12)
	Middle	**1.39**	**(0.60,3.22)**	0.47	(0.17,1.28)	0.91	(0.39,2.16)	1.22	(0.62,2.4)	0.99	(0.52,1.87)
High	Ref									
Insurance	Yes	1.62	(1.05,2.5)	**2.17**	**(1.28,3.70)**	1.36	(0.85,2.18)	0.76	(0.54,1.07)	0.82	(0.58,1.17)
	No	Ref									
Supplementary insurance	Yes	0.74	(0.53,1.05)	**0.17**	**(0.04,0.71)**	0.91	(0.62,1.34)	1.07	(0.81,1.43)	**1.35**	**(1.04,1.77)**
	No	Ref									
Concern about contracting COVID-19	Never	**0.58**	**(0.29,1.13)**	0.52	(0.19,1.49)	**0.43**	**(0.20,0.94)**	**0.42**	**(0.19,0.91)**	**0.80**	**(0.46,1.39)**
	No	**0.40**	**(0.25,0.65)**	0.50	(0.24,1.05)	**0.32**	**(0.18,0.57)**	**0.35**	**(0.20,0.61)**	**0.42**	**(0.29,0.62)**
	Yes	**0.68**	**(0.49,0.94)**	0.53	(0.31,0.91)	**0.59**	**(0.41,0.85)**	**0.61**	**(0.42,0.88)**	**1.02**	**(0.76,1.38)**
	Very concerned	Ref									
Hypertension	Yes	1.08	(0.56,2.08)	1.45	(0.58,3.6)	1.55	(0.80,3.03)	1.54	(0.83,2.85)	**1.83**	**(0.97,3.43)**
	No	Ref									
Asthma	Yes	1.7	(0.74,3.9)	1.51	(0.41,5.53)	1.13	(0.41,3.09)	4.25	(1.66,10.9)	**6.52**	**(1.91,22.3)**
	No	Ref									

EQ-5D-3L, EuroQol 5-Dimensional 3-Level questionnaire; OR, Odds Ratio; ref, Reference; Bold values are statistically significant, P < 0.05; ^*^Adjusted.

## Discussion

Participants' mean EQ-5D index and EQ-VAS scores were 0.80 and 77.53, respectively. Before the COVID-19 pandemic, two studies conducted on the general populations of Iran (based on a crosswalk methodology) (29) and South Australia (30) reported EQ-5D index scores of 0.79 and 0.91 and EQ-VAS scores of 71.7 and 78.5, respectively. Moreover, the mean EQ-5D index and EQ-VAS scores in studies conducted during the COVID-19 pandemic in China and Vietnam were 0.94 (85.5), and 0.95 (88.3), respectively (19, 22), which were higher than our results. Other studies in Portugal, Germany, Poland, Uruguay, and Italy before the COVID-19 pandemic reported scores of 0.86, 0.92, 0.89, 0.95, and 0.92, respectively (21, 31–34). It should be noted that we used the EQ-5D-3L value sets for Iran, and the EQ-5D value sets of each country are different. This issue may explain the difference between the results of the above studies and our research. Furthermore, the floor effect for the EQ-5D-3L in the Iranian study was lower than in other countries. Demographic characteristics such as female gender, older age, having a lower level of education, and having a lower income can justify the low HRQoL score in our study compared to the above studies.

According to our findings, higher utility scores were associated with a higher level of education. Previous research (28, 35–38) supported this result, while some studies demonstrated an inverse relation (19, 22). People with better education are more likely to have access to a healthy and clean environment, information and skills, and more financial resources.

Moreover, as in past studies (21, 36), employed subjects had significantly higher EQ-5D index values. However, other studies contradicted our results (19, 22). The COVID-19 pandemic has adversely affected the economy, and many people have lost their jobs as a result of their inability to obtain a minimum wage to support their families (39). The fear of economic loss has increased stress and caused psychological problems among people worldwide (39).

Similarly, income level was found to have a significantly positive relationship with HRQoL. It is evident that higher-income respondents are less concerned about living costs; therefore, they are expected to have higher utility scores. Despite our results, other studies conducted in the same COVID-19 period did not report a significant relationship between income level and utility scores (19, 22).

Consistent with our study, another research paper found a significant inverse correlation between the level of concern about contracting COVID-19 and utility scores (19). Fear of exposure to COVID-19, mental fatigue, insufficient information, financial damage, ambiguity in the disease's condition, and uncertainty about when the disease will end all cause stress and anxiety and affect the HRQoL of people during the pandemic.

Furthermore, in line with previous studies (19, 40, 41), there was a significant negative relationship between utility scores and hypertension, cerebrovascular disease, pulmonary disease, and asthma. The risk of severe COVID-19 increases among people with underlying medical conditions; this factor may make these people vulnerable and reduce their utility (42).

According to the findings, a large percentage of participants (44.2 and 53.7%) reported problems on the P/D and A/D dimensions, respectively. Before the COVID-19 crisis and using the same tool in the general population of Iran, these findings were confirmed by Goudarzi et al. (18). Similarly, several studies reported that the majority of complaints were on the P/D and A/D dimensions (19, 21, 28), while Saarni et al. (43) and König et al. (44) found the most issues on the P/D and MO dimensions.

Multiple logistic regression revealed that higher age groups, lower income levels, and concern about getting COVID-19 increased the likelihood of reporting problems on the MO dimension significantly. Ping et al. reported similar results, only about the impact of age (19), while a study on Palestinians found no significant relationship between the chance of reporting a problem on the MO dimension and demographic characteristics (45).

Regression analysis also indicated that the likelihood of reporting a problem on the SC dimension was considerably associated with age, employment, and insurance. Similar results for the impact of employment were reported by Ping et al. (19) in China. Hamdan et al. also reported a significant association between the probability of reporting a problem in SC and age (45).

Additionally, we found that the probability of reporting a problem on the UA dimension increased significantly with being a male individual, aging, and concerned about getting COVID-19. Hamdan et al. discovered that participants with a college education were significantly less likely to report problems in the UA than those with a high school education (45). A study conducted in India found a significant relationship between gender and place of residence and the likelihood of reporting problems on the UA dimension (46).

Similar to the study in China (19), this research showed that the odds of reporting a problem on the P/D dimension increased significantly with age and concern about getting COVID-19. Furthermore, our results showed that the probability of reporting a problem on the A/D dimension was significantly higher in female interviewees, in the unemployed, in participants with supplementary insurance, in people who were worried about getting COVID-19, and in those with hypertension and asthma.

### Public health implications

The findings of this study can be used to identify the unmet health needs of the population, recognize inequalities and determinants of population health, help policymakers and health planners make informed decisions and develop healthcare programs, and can also be used to evaluate public health programs and ensure that the population benefits from these programs.

### Strengths and limitations

This is the first study in Iran to analyze HRQoL and its predictors among the general population during the COVID-19 pandemic. Having a sufficient sample size was another strength of the current study. Despite these advantages, our study has some limitations. To begin with, data collection *via* an online questionnaire (web survey) may be subject to selection bias some people such as the illiterate, the elderly, and those with low socioeconomic status. Thus, the first limitation is associated with its generalizability to the whole Iranian community. Secondly, the convenience sampling method has been used in this research, which cannot be fully representative of the population because the samples are not selected at random. Also, cross-sectional studies cannot demonstrate a causal relationship. Furthermore, EQ-5D-3L has higher ceiling effects than EQ-5D-5L. Therefore, the results of this study should be interpreted with caution. In addition to the above, the use of the OR is a limitation because it tends to overestimate the measure of association when compared to the use of the prevalence ratio. Finally, worry about getting COVID-19 was assessed by one question in this study, whereas standard instruments to measure such psychological distress have been developed by Ahorsu et al. (47) and Taylor et al. (48). As a result, it is necessary to use a valid and standardized instrument to assess the impact of COVID-19 on mental health in future studies.

## Conclusions

This study provides crucial insights into HRQoL and its influencing factors among the Iranian general population during the COVID-19 pandemic, which will be useful to policymakers. Indeed, accurate knowledge of community health helps planners and policymakers in their decision-making. The risk of P/D increased significantly among people who were aging and concerned about contracting COVID-19. The risk of A/D also increased significantly among men, in addition to those participants with hypertension and asthma, those who were unemployed, those with insurance, and those who were concerned about getting COVID-19. In all age groups, more than half of the participants are affected by A/D. Therefore, during pandemics, the mental health of people, especially those with chronic diseases, should be considered. The implementation of psychological counseling programs and medical interventions is needed to improve population health.

## Data availability statement

The raw data supporting the conclusions of this article will be made available by the authors, without undue reservation.

## Ethics statement

The studies involving human participants were reviewed and approved by the Committee of Ethics of the Tehran University of Medical Sciences IR.TUMS.MEDICINE.REC.1399.434. The patients/participants provided their written informed consent to participate in this study.

## Author contributions

MSS, SE, AAS, and HK contributed to the design and conception of the study. MSS and HK organized and prepared data file, wrote the first draft of the manuscript, co-supervised the research, and data acquisition. MSS, MT, HK, and HA performed statistical analysis. MSS, HK, HA, and SA wrote the sections of manuscript. SE and AAS supervised the study. SE, AAS, and HA commented on the manuscript. All authors contributed in manuscript revision, proofread, and approved the submitted version.
